# Pathogenesis of Brain Edema and Investigation into Anti-Edema Drugs

**DOI:** 10.3390/ijms16059949

**Published:** 2015-04-30

**Authors:** Shotaro Michinaga, Yutaka Koyama

**Affiliations:** Laboratory of Pharmacology, Faculty of Pharmacy, Osaka Ohtani University, Tonda-bayashi, Osaka 584-8540, Japan; E-Mail: mitinasy@osaka-ohtani.ac.jp

**Keywords:** aquaporin, blood-brain barrier, cold injury, cytotoxic edema, ET_B_ receptor, fluid percussion injury, matrix metalloproteinase, vascular endothelial growth factor, vasogenic edema

## Abstract

Brain edema is a potentially fatal pathological state that occurs after brain injuries such as stroke and head trauma. In the edematous brain, excess accumulation of extracellular fluid results in elevation of intracranial pressure, leading to impaired nerve function. Despite the seriousness of brain edema, only symptomatic treatments to remove edema fluid are currently available. Thus, the development of novel anti-edema drugs is required. The pathogenesis of brain edema is classified as vasogenic or cytotoxic edema. Vasogenic edema is defined as extracellular accumulation of fluid resulting from disruption of the blood-brain barrier (BBB) and extravasations of serum proteins, while cytotoxic edema is characterized by cell swelling caused by intracellular accumulation of fluid. Various experimental animal models are often used to investigate mechanisms underlying brain edema. Many soluble factors and functional molecules have been confirmed to induce BBB disruption or cell swelling and drugs targeted to these factors are expected to have anti-edema effects. In this review, we discuss the mechanisms and involvement of factors that induce brain edema formation, and the possibility of anti-edema drugs targeting them.

## 1. Introduction

Brain edema is a fatal pathological state in which brain volume increases as a result of abnormal accumulation of fluid within the cerebral parenchyma [1]. The abnormal accumulation of fluid causes an increase on brain volume and elevation of intracranial pressure (ICP) because of an enclosed rigid skull. The increase in brain volume results from an increase in brain components including cerebral tissue, blood and cerebrospinal fluid (CSF) compartments, and is observed prior to elevation of ICP [2,3]. The increased ICP is caused by the increased brain volume, and the relationships between brain volume and ICP are shown as exponential but not linear one [2,3]. The elevation of ICP in the brain induces adverse conditions including reduction of cerebral blood, hypoxia and pressure of the cerebral tissue and hernia. These, in turn, cause an irreversible impairment of nerve function, and at worst, death. Thus, the severity of brain edema is correlated to the increased ICP. Brain edema has been observed in head trauma, cerebral ischemia, hemorrhage and liver failure [4,5,6,7], and delays in recovery after brain damage. Despite the serious pathogenesis of brain edema, medical strategies are limited. Although symptomatic treatments such as corticosteroids and hypertonic solutions have been conducted [8,9,10], the therapeutic effects are insufficient because these medicines cannot remove fundamental causative factors or be used for a long period because of their adverse side effects. Thus, the development of novel anti-edema drugs is required. Because the pathogenesis of brain edema is complicated, understanding the detailed mechanisms of brain edema formation is essential for the development of anti-edema drugs. Using experimental animal models of brain edema, various key molecules have been found to be involved, and subsequently the effects of candidate drugs have also been studied in these animals. In this review, we focus on several key factors, summarize effective anti-edema drugs reported in experimental animal models, and consider novel therapies for brain edema.

## 2. Classification of Brain Edema

**Figure 1:**
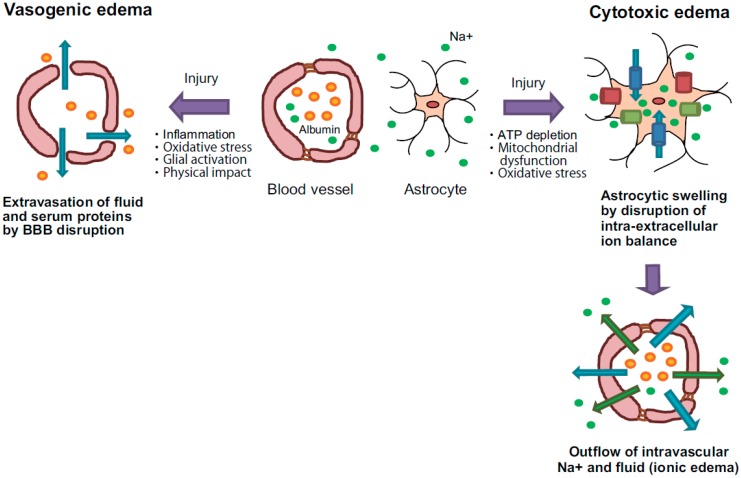
Pathology of vasogenic and cytotoxic edema. Vasogenic edema: After brain injuries, endothelial tight junctions are disrupted by inflammatory reactions and oxidative stress. Moreover, activated glial cells release vascular permeability factors and inflammatory factors, and these factors accelerate blood-brain barrier (BBB) hyperpermeability. These events cause extravasation of fluid and albumin, leading to extracellular accumulation of fluid into the cerebral parenchyma. Cytotoxic edema: Brain insults induce intracellular ATP depletion, resulting in mitochondrial dysfunction and oxidative stress. These events cause a disturbance of intra-extracellular ion balance. As a result, excessive inflows of extracellular fluid and Na^+^ into cells are induced, leading to cell swelling. Because the extracellular Na^+^ contents are decreased by excessive inflow into cells, the outflow of Na^+^ and fluid from blood vessels is compensatorily accelerated. The intravascular Na^+^ outflow results in extracellular fluid accumulation in the cerebral parenchyma. Blue arrows: flow of water, green arrows: flow of Na^+^, orange spheres: albumin, green spheres: Na^+^, blue columns: water channel, green columns: ion transporter and red columns: ion channel.

Brain edema is mainly classified into vasogenic edema and cytotoxic edema. Vasogenic edema is characterized by extravasation and extracellular accumulation of fluid into the cerebral parenchyma caused by disruption of the blood-brain barrier (BBB) (Figure 1). In contrast, cytotoxic edema is characterized by intracellular accumulation of fluid and Na^+^ resulting in cell swelling (Figure 1). After the formation of cytotoxic edema, extravasation of fluid is evoked by disruption of the osmotic pressure gradient resulting from decreased extracellular Na^+^ without BBB disruption (ionic edema). In clinical pathophysiology of brain injury, the time windows of formation and recovery in vasogenic edema and cytotoxic edema are different [5,11]. After ischemic stroke, cytotoxic edema is first observed within a few hours and then declines within 1 day. Conversely, vasogenic edema forms within two to three days and is maintained for several days. In this section, the mechanisms of vasogenic and cytotoxic edema are discussed.

### 2.1. Vasogenic Edema

Vasogenic edema is due to BBB disruption, resulting in extravasation of fluid and intravascular proteins such as albumin into the cerebral parenchyma (Figure 1). The extravasated fluid accumulates outside the cells, and the excessive extracellular accumulation of fluid evokes an increase of brain volume and ICP.

To protect from extravasation of serum components and the import of extraneous substances into the cerebral parenchyma, cerebral blood vessels have a particular structure that is different from peripheral tissues. Brain endothelial cells reciprocally connect and constitute tight junctions through extracellular adhesion proteins and form BBB with astrocytes and pericytes [12,13,14]. Brain insult-induced reversible and irreversible BBB disruptions cause vasogenic edema. After brain injuries, temporal ischemia reperfusion causes excitotoxicity and oxidative stress through mitochondrial dysfunction [15,16,17]. These events may directly damage BBB-constituting cells, resulting in irreversible BBB disruption. Moreover, the ischemia reperfusion induces migration of leucocytes [18] and activation of glial cells such as microglia and astrocytes [19]. These cells excessively release vascular permeability factors, cytokines and chemokines, leading to BBB hyperpermeability [14,20,21]. Because BBB disruption is reversible, it may be possible to recover using medicine. BBB disruption and vasogenic edema are commonly observed in cerebral trauma, hemorrhage and the secondary phase of ischemia [4,5,6]. Thus, the recovery of reversible BBB disruption may be possible for these types of injury.

### 2.2. Cytotoxic Edema

Cytotoxic edema is characterized by abnormal accumulation of fluid into brain cells and cell swelling (Figure 1), and is commonly observed in cerebral ischemia and liver failure [22,23].

In cerebral ischemia, ATP depletion and disturbance of intra-extracellular Na^+^ transportation are responsible for cytotoxic edema formation [22,24]. The glucose supply for brain cells is remarkably diminished by deterioration of the brain blood flow after cerebral ischemia, which causes a decrease of intracellular ATP production. The ATP depletion induces a failure of intra-extracellular Na^+^ transport systems and excessive intracellular Na^+^ accumulation. The increase of intracellular Na^+^ leads to an abnormal entry of extracellular fluid into cells, resulting in cell swelling.

In acute liver failure, several deleterious products such as ammonia, which are normally removed by hepatic metabolism, accumulate in various tissues including the brain. In central nervous tissue, parts of these products are taken into astrocytes and cause oxidative stress and mitochondrial dysfunction [23]. These events lead to astrocytic dysfunction and swelling.

After cytotoxic edema formation, the outflow of Na^+^ from blood vessels is accelerated as the body tries to improve decreases of extracellular Na^+^ and fluid [22]. The intravascular Na^+^ outflow induces an extravasation of fluid without BBB disruption, and causes extracellular fluid accumulation known as ionic edema (Figure 1). Thus, cytotoxic edema also causes an increase of brain volume and ICP. These observations indicate that anti-cytotoxic edema drugs are also expected to improve the abnormal outflow of intravascular fluid and increased ICP after brain insult.

## 3. Experimental Models of Brain Edema in Animals

To elucidate the effects of candidate anti-edema drugs, various experimental animal models have been adopted. As described above, the mechanisms of vasogenic edema and cytotoxic edema are different. Thus, the choice of experimental models reflects each edema and should be taken into consideration when examining the effects of anti-vasogenic or anti-cytotoxic edema drugs. In this section, the relevance of experimental models in relation to vasogenic or cytotoxic edema is discussed.

### 3.1. The Cold Injury Model

**Figure 2:**
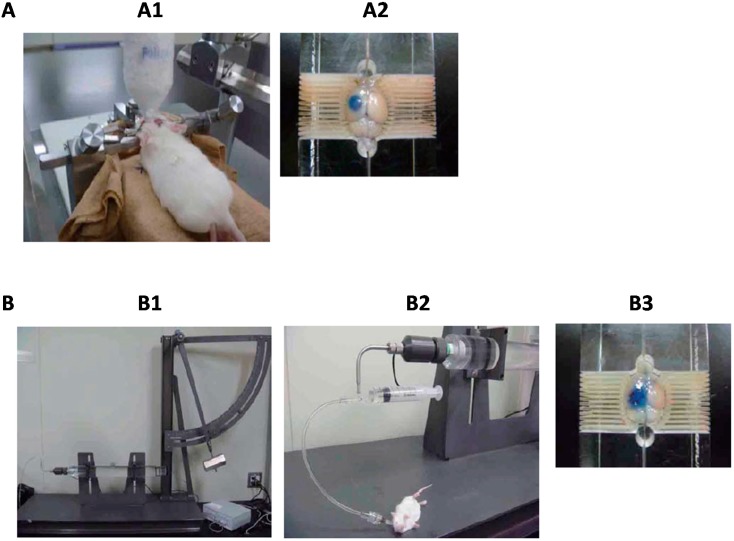
Brain edema model in experimental animals. (**A**) Cold injury is performed by inflicting freeze stimulation on the hemisphere of the skull of the animal (**A1**). After cold injury, BBB disruption is indicated by evaluating extravasation of Evans blue dye (**A2**); (**B**) Fluid percussion injury is performed by an injury to the intact dura after craniectomy by impacts of rapidly pushed fluid (**B1**,**B2**). As well as cold injury, the extravasation of Evans blue dye is observed (**B3**).

The cold injury model is performed by inflicting freeze stimulation on the hemisphere of the skull of the animal (Figure 2A). The freezing and thawing of central nerve tissues by cold injury directly impairs the integrity of vascular endothelial cells and enhances extravasation of intravascular proteins through disrupted BBB [25,26,27]. The cold injury model is therefore mainly adopted as an experimental model of vasogenic brain edema. In core and peri-core areas after cold injury, the mechanisms and time windows of vascular hyperpermeability are different. The cold injury directly damages vascular cells, resulting in irreversible BBB dysfunction in the core area. Conversely, different mechanisms are responsible for BBB disruption in the peri-core area. Nitric oxide and proinflammatory peptides such as kinins have been indicated to be involved in BBB disruption because synthesis inhibitors and receptor antagonists showed less BBB hyperpermeability and edema formation after cold injury [25,28]. In addition, cold injury induced activation of glial cells such as astrocytes and microglia around the lesioned area and caused glial inflammatory responses [29]. These inflammatory responses also exacerbated the cold injury-induced brain edema because of BBB breakdown. Although the cold injury-induced brain edema is unlike typical clinical brain edema when compared with brain edema in other experimental models, there are beneficial points such as experimental repeatability and clarity of injury area. We also confirmed cold injury-induced brain edema formation and BBB disruption by extravasation of Evans blue dye in mice (Figure 2A).

### 3.2. The Fluid Percussion Injury (FPI) Model

Traumatic brain injury (TBI) induces focal cerebral damage by physical impact in core areas, and subsequently, secondary injury is evoked, resulting in diffuse cerebral damage in the peri-core area. The secondary injury persistently causes BBB disruption, brain edema and neuronal degeneration in diffuse cerebral areas [30,31,32,33]. Thus, treating the secondary injury is essential for alleviating TBI damage. To elucidate the mechanisms and examine the effects of candidate therapeutic agents for TBI-induced secondary injury, various models of TBI including FPI, weight drop impact and cortical impact have been developed [34,35].

FPI is an experimental model of human TBI in animals and causes an injury to the intact dura after craniectomy by the impact of rapidly pushed fluid [36,37]. A representative device for FPI is shown in Figure 2B. Based on the position of the craniotomy away from the sagittal suture, FPI models are mainly categorized into central (centered on the sagittal suture) and lateral models (>3.5 mm lateral to midline) [36]. Both FPI models induce focal and diffuse injury, resulting in cerebral edema. Vasogenic and cytotoxic edema have been observed in FPI animal models [38]. The FPI-induced BBB disruption and hyperpermeability were indicated in the injured cerebrum [39,40,41,42,43]. In accordance with these reports, an extravasation of Evans blue dye was also observed in the core and peri-core area after FPI (Figure 2B). Similar to the cold injury model, FPI also causes an irreversible BBB disruption by physical impact in the core area, and the diffuse injury induces a secondary BBB disruption by different mechanisms in the peri-core area. FPI induces activation of various catabolic enzymes such as matrixmetalloproteinase-9 and causes degradation of vascular basal lamina, resulting in BBB breakdown [43]. The involvement of inflammatory responses for FPI-induced BBB disruption and secondary injury processes are also implicated because an FPI-induced increase of inflammatory mediators and infiltration of macrophages are observed [44,45,46].

Cytotoxic edema develops early and persists after TBI [4], and the development of cytotoxic edema has also been indicated in FPI animal models [47]. FPI induces the alteration of cellular membrane transporters and channels involved in transporting fluid and Na^+^, and causes cell swelling. Because the FPI model fully reflects TBI-induced cerebral edema, this model has been commonly adopted for evaluating the effects of candidate anti-edema drugs.

### 3.3. The Cerebral Hemorrhage Model

Intracerebral hemorrhage (ICH) and subarachnoid hemorrhage (SAH) are lethal conditions characterized by outflow of circulating blood into the cerebral parenchyma or subarachnoid space, respectively [48,49,50]. After initial hemorrhage, continued bleeding is observed and hematoma expansion is induced, which is consequently associated with adverse outcomes. In the area surrounding hematoma, secondary injury is induced by the disturbance of neuronal and glial functions. These events cause glutamate release, membrane depolarization and mitochondrial dysfunction [49]. Severe mitochondrial dysfunction leads to cellular swelling. In addition, because activated glia release products that induce BBB breakdown, BBB dysfunction is deteriorated, resulting in extravasation of blood components (e.g., thrombin and hemoglobin) and inflammatory responses [49,51,52,53,54]. Thus, both vasogenic and cytotoxic edema are observed after hemorrhage. Because brain edema causes the mortal outcome after hemorrhage [48,55,56], anti-edema drugs are required for therapy of hemorrhage.

The experimental ICH model damages vessels by injection of collagenase, which disrupts the basal lamina of blood vessels, or injection of autologous blood into the brain parenchyma [52,57,58]. In the SAH model, single-hemorrhage, double-hemorrhage and endovascular puncture models have been commonly made [59]. The single-hemorrhage model is made by injecting fresh syngeneic arterial blood into the cisterna magna. In the double-hemorrhage model, two injections with autologous arterial blood are given. In the endovascular puncture model, a suture is set in the external carotid artery and threaded through the internal carotid artery up to the middle cerebral artery. In this area, the vessel is punctured, resulting in hemorrhage. Because these models induce brain edema similar to clinical hemorrhage, several candidate anti-edema drugs have been studied in these models.

### 3.4. The Water Intoxication Model

The intra-extracellular water balance depends on Na^+^ conditions, and the fluctuation of intra-extracellular Na^+^ contents leads to water inflow into cells or water outflow from cells. Disturbance of the Na^+^ balance as seen in hyponatremia (low levels of Na^+^) induces disruption of the water balance. In clinical situations, hyponatremia is induced by antidiuretic hormone (ADH) secretion abnormality, renal diseases and excessive water intake. Hyponatremia causes a decrease of extracellular Na^+^ contents and relative increase of intracellular Na^+^ contents. Subsequently, water inflow into cells is accelerated. In hyponatremia patients, cell swelling and brain edema have been observed [60], but BBB damages have not been observed. Thus, cytotoxic edema but not vasogenic edema is predominant in hyponatremia-induced brain edema. Because the hyponatremia-induced brain edema is a dangerous condition for the induction of herniation, attenuation of brain edema may be a beneficial therapy.

In experimental animal models, the water intoxication model best reflects simulation of hyponatremia because it induces a relative decrease of extracellular Na^+^ concentration. The water intoxication model is produced by intraperitoneal loading of excessive distilled water corresponding to 10%–40% of the body weight of experimental animals [61,62,63,64,65,66]. The excessive loading of water induces an increase of water content in central nerve tissue and an excessive influx of water into astrocytes, resulting in astrocytic swelling [63,64,65,66]. Because brain cell swelling but not BBB damage is predominantly observed in water-intoxicated animals, this has been adopted as a model of cytotoxic edema.

### 3.5. The Liver Failure Model

Liver failure results from acute or chronic dysfunctions of hepatic cells and induces hepatic encephalopathy, causing severe dysfunction in central nerve tissue. Although brain edema is a common feature in acute and chronic liver failure, the pathogenesis of brain edema is different. In acute liver failure, an increase of ICP is observed resulting in brain herniation [2,67]. On the other hands, the increase of ICP is rarely observed in chronic liver disease [2,68]. These discrepancies could be explained due to differences of volume in brain components including brain tissue, blood and CSF after liver failure or to age-related atrophy [2].

In the liver failure model, astrocytic swelling is observed and cytotoxic edema formation is indicated [7,69,70,71,72,73]. Although ammonia is normally removed by hepatic metabolism, it is accumulated under hepatic dysfunction. Ammonia is one of the key inducers and mechanisms of cytotoxic edema by liver failure [74,75,76]. Astrocyte swelling has been observed in experimental models of hyperammonemia and treatment with ammonia-induced astrocytic swelling in cultured astrocytes [75]. In central nervous tissue, ammonia is taken into astrocytes and converted to glutamine with glutamate. Glutamine is indicated to be responsible for ammonia-induced hepatic encephalopathy. Because excessive glutamine induces oxidative stress and mitochondrial dysfunction [77,78], these events may be involved in ammonia-induced astrocytic swelling. Meanwhile, vasogenic edema is not predominant in liver failure-induced edema because BBB damages are not observed. Thus, anti-cytotoxic edema drugs must be beneficial for cerebral edema in liver failure.

To reflect human liver failure-induced brain edema in animals, experimental animals are commonly treated with thioacetamide, which induces critical damage to hepatocytes [79,80,81,82,83]. In this model, astrocytic swelling is predominantly observed as well as liver failure [79,80,81,82,83]. Moreover, treatment of galactosamine in animals has been performed for inducing acute liver failure [2,84,85], and treatments of bile duct-ligation or portacaval anastomosishas have been performed for inducing chronic liver failure [2,73,86]. Several studies suggest that ammonia induces fluctuations of astrocytic membrane water channels and cation transporter functions [87,88]. Because these channels and transporters play a key role in intra-extracellular balances of water and Na^+^, respectively, ammonia-induced disturbance of these channels would induce an excessive entry of water and Na^+^ into cells and lead to cell swelling after liver failure.

## 4. Methods for Evaluating Brain Edema

### 4.1. Wet-Dry Weight Method

The wet-dry weight method is a common and simple method for evaluating brain edema after brain insults in experimental animals. It is invasive and not performed in patients. This method is based on the weight measurement of brain tissue before and after complete dehydration [2]. The weight before dehydration is shown as “wet weight” and the weight after dehydration is shown as “dry weight”. After measurement of wet and dry weight, the brain water content and tissue swelling can be calculated by the below equations [89].

Water content (%) = (wet weight − dry weight) × 100/wet weight(1)

Water content = (wet weight − dry weight)/dry weight(2)

Tissue swelling (%) = (final wet weight − initial wet weight) × 100/initial wet weight(3)

### 4.2. The Gravimetric Method

The gravimetric technique is based on calculating the percentage of water from measuring the density of the tissue in experimental animals [2,90]. This method is also invasive and not performed in patients. The specific brain area such as cortex and white matter from freshly dissected brain tissue are dropped into linear density gradient columns, and the density of the brain tissue is measured and the percentage of water is determined [2,90]. The gravimetric technique has several advantages including higher sensitivity and use of smaller pieces of tissue over the wet-dry weight method [2].

### 4.3. Magnetic Resonance Imaging (MRI)

As a noninvasive method, MRI has been used for evaluating brain edema in patients and experimental animals. By apparent diffusion coefficient (ADC) and T2 imaging, cytotoxic and vasogenic edema after brain injury can be assessed [91,92]. ADC is an indicator for magnitude of diffusion of water molecules within tissue and the diffusion imaging provides information about cellular architecture such as cellular size, membranes and volume fraction [93]. Because an extracellular space within cerebral parenchyma becomes narrower when cell swelling is observed, the ADC value is reduced [93]. T2 value is a transverse relaxation time of excited protons and the weighted image is basic pulse sequence in MRI. T2 signal intensity is related to increased vascular permeability and water content. In general, the reduced ADC values correlate with cytotoxic edema, whereas the increased T2 values reflect the development of vasogenic edema [91,92,93].

## 5. Key Molecules of Brain Edema Formation: Possible Targets of Anti-Edema Drugs

**Figure 3:**
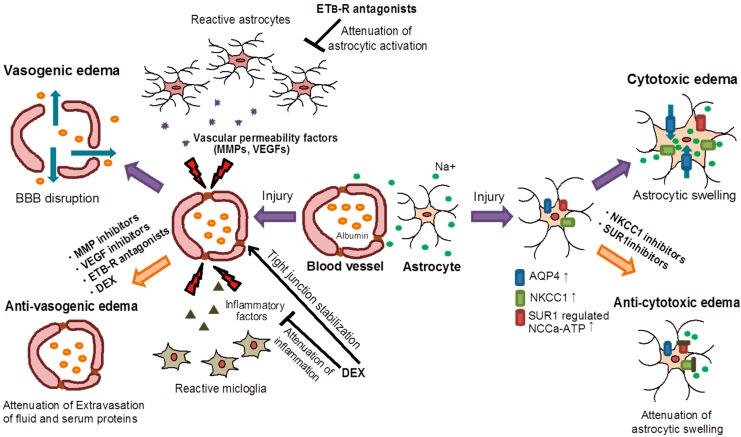
Involvement of key molecules and effects of candidate anti-edema drugs in brain edema. After brain insult, matrixmetalloproteinases (MMPs) and vascular endothelial growth factors (VEGFs) are increased, causing disruption of tight junctions and BBB hyperpermeability. ETB-R antagonists attenuate the activation of astrocytes and production of MMPs and VEGFs. Dexamethasone (DEX) reduces the production of inflammatory factors and accelerates the stabilization of tight junctions. Thus, these agents are expected to be anti-vasogenic edema drugs. Brain insults induce dysfunctions of aquaporin 4 (AQP4), Na^+^–K^+^–Cl^−^–Co-Transporter 1 (KNCC1) and (sulfonylurea receptor 1) SUR1-regulated conselective cation channels (NC_Ca-ATP_). These dysfunctions induce disruption of intra-extracellular Na^+^ and water balance, resulting in cell swelling. Thus, agents affecting AQP4, KNCC1 and SUR1-regulated NC_Ca-ATP_ would be beneficial for cytotoxic edema. Blue arrows: flow of water, green arrows: flow of Na^+^, orange spheres: albumin, green spheres: Na^+^, purple spheres: vascular permeability factors, brown triangle: inflammatory factors.

A variety of molecules including vascular permeability factors, membrane channels, transporters and receptors are known to be responsible for brain insult-induced vasogenic and cytotoxic edema (Figure 3). In recent studies, these molecules have been the target and focus for anti-edema drugs, some of which are described in this section.

### 5.1. Vascular Endothelial Growth Factors (VEGFs)

VEGFs are common angiogenic factors and induce the proliferation and migration of vascular endothelial cells in various tissues including the brain [94]. In central nervous tissue, the production of VEGFs is observed in astrocytes, neurons and endothelial cells [95,96,97,98,99,100]. An increase in VEGFs is seen in patients after brain insult [101,102,103]. Similar findings were also indicated in cold injury [1], FPI [104,105] and SAH experimental animal models [106].

In addition to their angiogenic effects, VEGFs are also known to enhance BBB permeability [21,107,108,109,110]. The barrier functions of the BBB are dependent on the function of brain microvessel endothelial cells (BMVECs), which constitute tight junctions and restrict vascular permeability [12]. Claudins (CLNs) and occludin (OCLN) are transmembrane proteins regulating tight junctions. BMVECs predominantly express CLN-5 and the CLN-5 integrates tight junction properties [111]. Because CLN-5^−/−^ mice display selective BBB openings, CLN-5 is essential for the restriction of BBB permeability [112]. As well as CLN-5, OCLN also contributes to junction properties and regulates BBB permeability [113]. As one of the main mechanisms of VEGF-induced BBB hyperpermeability, the effects for CLN-5 and OCLN expressions have been reported. The treatment of VEGF-A induced down-regulation of CLN-5 and OCLN in human BMVECs [21]. Similarly, decreases in CLN-5 and OCLN were also observed in mice cerebrum when VEGF-A was administered [21]. These findings indicate that VEGF induces BBB hyperpermeability by disrupting tight junction-regulating proteins.

The effects of VEGF antagonism have been examined in experimental animals with brain injury. In cerebral ischemia when rats were administered VEGF neutralizing antibody, the vasogenic edema area was reduced as indicated by MRI [114]. Similarly, mFlt (1–3), which sequesters murine VEGF, led to a significant reduction in the volume of the edematous tissue in cerebral ischemia mice [115]. In rats administered gene transfer of soluble flt-1 (sFlt-1), a natural inhibitor of VEGF, ischemia-induced BBB hyperpermeability evaluated by extravasation of Evans blue dye was attenuated, and the size of the brain edema was smaller than in vehicle-administered rats [116]. Moreover, the VEGF receptor antagonist VGA1155 reversed the increase of brain water content and attenuated BBB disruption in cold injury rats [117]. Thus, drugs that antagonize VEGF are expected to be beneficial for vasogenic edema.

### 5.2. Matrixmetalloproteinases (MMPs)

MMPs are a family of zinc-endopeptidases responsible for the degradation of extracellular matrix molecules such as collagen, laminin and fibronectin [118]. Although MMPs support the repair of damaged nerve tissues by promoting angiogenesis as VEGFs do [119], their excessive action disrupts the integrity of vascular endothelial cells by degrading the basal lamina around brain microvessels, resulting in BBB hyperpermeability [120]. In patients with brain injury, up-regulation of MMPs has been indicated along with the severity of brain injury [102,121,122,123]. In central nervous tissue, the production of MMPs, especially MMP9, was observed in astrocytes, microglia, neurons and endothelial cells, and the increased MMP expression and activity were confirmed in experimental animals after brain insult [124,125,126,127,128,129,130,131,132]. Because cerebral ischemia-induced BBB disruption was significantly attenuated in MMP9 knock-out mice [133], MMP9 is indicated as a key inducer of BBB disruption after brain insult.

In experimental animals with brain injury, the effectivity of MMP inhibitors for brain edema has been examined. The administration of GM6001, a broad-spectrum MMP inhibitor, reduced injury volume and brain water contents in intracerebral hemorrhage mice [134]. Similar observations were made in traumatic brain injury in rats [135]. Moreover, other broad-spectrum MMP inhibitors such as BB-1101 and MMI270 also reduced BBB hyperpermeability and brain edema in experimental animals with intracerebral hemorrhage [125], cerebral ischemia [136,137] and cold injury [138]. These results suggest that MMP inhibitors are prime candidates for anti-vasogenic edema drugs.

### 5.3. Aquaporins (AQPs)

AQPs are major water channel proteins for fluid transportation across plasma membranes and they regulate intra-extracellular water balance. At least 13 subtypes of AQPs have been identified and the expression of AQP4 is the most abundantly observed in central nervous tissue, although AQP1 and AQP9 have also been reported [1,61,139,140]. AQP4 is predominantly expressed in astrocyte foot processes surrounding capillaries known as end-feet, and it plays a key role in brain water balance by regulating water fluxes into and out of the brain parenchyma [1,61,140,141]. The expression of AQP1 is predominantly observed in the choroid plexus and is involved in cerebrospinal fluid (CSF) formation [142]. AQP9 protein has been detected weakly by antibody staining in some astrocyte processes at the glia limitans [143]. In studies of brain edema, AQP4 has been extensively studied.

AQP4 expression is known to fluctuate under various brain insults. In patients with traumatic brain injury, increased AQP4 expression was observed [144]. In experimental animals, the fluctuation of AQP4 expression is complicated. Increased AQP4 was observed in FPI [145], ICH [146] and liver failure animals [81]. Conversely, several studies show a decrease in AQP4 in other traumatic brain injury [147] and cerebral ischemia mice [148]. Evidence that AQP4 is responsible for cytotoxic brain edema is indicated by studies of AQP4-null mice, where AQP4 deletion reduced cytotoxic brain edema in water intoxication and ischemic stroke mice [63]. Similar findings were observed in liver failure animals [76]. However, the involvement of AQP4 is considerably complicated in the pathogenesis of brain edema. Contrary results have been observed in the vasogenic edema model. AQP4 null mice were observed to have more severe brain edema compared with wild-type mice in cortical cold injury [149] and intracerebral hemorrhage [150]. Additionally, the elimination of intraparenchymal fluid was slower in AQP4-null mice compared with wild-type mice, suggesting that the vasogenic edema-derived accumulation of cerebral fluid into parenchyma is mainly eliminated by an AQP4-dependent route [61]. In summary, AQP4 has opposing roles in the pathogenesis of cytotoxic and vasogenic edema. In cytotoxic edema, AQP4 induces the excessive influx of extracellular fluid into cells, while AQP4 is involved in the excretion of fluid accumulation into the parenchyma in vasogenic edema. Thus, AQP4 inhibitors are expected to be beneficial for cytotoxic edema by attenuating cell swelling, while AQP4 activators or up-regulators may be effective in vasogenic edema, resulting in facilitation of fluid clearance in the parenchyma. Recently, 2-(nicotinamide)-1,3,4-thiadiazole (TGN-020) was identified as a novel aquaporin 4 (AQP4) inhibitor and administration of TGF-020 reduced ischemic cerebral edema in mice [151]. Moreover, Piroxicam, a nonsteroidal anti-inflammatory drug (NSAID) also exerted an AQP4 inhibitory action by binding to AQP4 and reduced cerebral edema formation in rodent cerebral ischemic model [152]. Thus, these AQP4 inhibitors are expected to show beneficial effects for clinical brain edema in future.

### 5.4. Na^+^–K^+^–Cl^−^–Co-Transporter 1 (NKCC1)

NKCC1 transports sodium and potassium with chloride into and out of cells, and plays an important role in the maintenance of physiological intra-extracellular Na^+^ concentration levels and regulating cell volume [22,24,153]. In central nervous tissue, NKCC1 expression is observed in astrocytes and endothelial cells [22,24,153]. NKCC1 function is known to be disturbed in pathological situations. After traumatic brain injury and cerebral ischemia in experimental animals, increased NKCC1 expression was induced and caused an excessive inflow of extracellular Na^+^ into cells [22,24,153,154,155,156]. Moreover, evidence for the involvement of NKCC1 in brain edema formation is indicated in NKCC1-null mice. NKCC1-null mice exhibited no cell swelling and less severe cerebral ischemia-induced brain edema compared with wild-type mice [157,158]. Thus, NKCC1 is a key inducer for cytotoxic edema formation.

Bumetanide is an inhibitor of NKCC1 and is used clinically as a loop system diuretic agent. In experimental animals, the effects of bumetanide on brain edema have been confirmed. The treatment of bumetanide has been shown to attenuate cytotoxic edema induced by cerebral ischemia [153,159], traumatic brain injury [160] and liver failure [161]. These results suggest that bumetanide is expected to be a candidate for anti-cytotoxic edema.

### 5.5. Sulfonylurea Receptor 1 (SUR1)-Regulated Nonselective Cation Channels (NC_Ca-ATP_)

SUR1-regulated NC_Ca-ATP_ is a nonselective cation channel whose function is regulated by intracellular calcium and ATP [162,163]. Although SUR1-regulated NC_Ca-ATP_ is absent in physiological states, expression is confirmed in experimental animals after brain injury [163,164]. Following traumatic brain injury and cerebral ischemia in animals, the up-regulation of SUR1-regulated NC_Ca-ATP_ was observed in astrocytes, neurons, and capillaries [42,163,165,166]. The opening of SUR1-regulated NC_Ca-ATP_ is evoked by the depletion of intracellular ATP and induces an excessive inflow of Na^+^ into cells, causing cytotoxic edema [155,163].

SUR1 is sensitive to sulfonylurea inhibitors such as glibenclamide, which is used as an anti-diabetic agent. In cerebral ischemia and traumatic brain injury animals, treatment with glibenclamide showed a significant reduction in the development of brain edema [166,167,168]. These findings indicate that SUR-1-regulated NC_Ca-ATP_ is crucially responsible for the development of cytotoxic edema after brain insults, and that glibenclamide is expected to provide a new therapeutic approach to cytotoxic edema.

### 5.6. Endothelin ET_B_ Receptor (ET_B_-R)

Endothelins (ETs) were intrinsically discovered as vasoconstrictor peptides and have multiple physiological actions other than vascular constriction in nonvascular tissues, including central nervous tissue [169,170,171]. ET receptors have two distinct types: ET_A_ receptor (ET_A_-R) and ET_B_ receptor (ET_B_-R). In central nervous tissue, these cellular distributions and functions are different. ET_A_-R is present in vascular smooth muscle and the ET_A_-R activation induces vasoconstriction [172]. ET_B_-R is predominantly observed in astrocytes [173,174,175]. ET_B_-R activation in resting astrocytes induces phenotypic conversion to reactive astrocytes and stimulates several pathophysiological responses [176,177]. In the rat cerebrum and cultured astrocytes, MMP9 and VEGF-A were increased by ETs acting through ET_B_-R [178,179,180]. These observations imply that the production of astrocytic MMP9 and VEGF-A is regulated by ET_B_-R.

We previously examined the effects of selective ET_B_-R antagonists (BQ788 and IRL-2500) on brain edema formation in cold injury mice. Intracerebroventricular administration of BQ788 and IRL-2500 attenuated cold injury-induced BBB disruption and vasogenic brain edema [181]. In this study, BQ788 and IRL-2500 also reduced a cold injury-induced increase of reactive astrocytes [181]. Because reactive astrocytes produce various vascular permeability factors including MMP9 and VEGF-A, the reduction of reactive astrocytes may be beneficial for BBB disruption. In agreement with our findings, ET_B_-R antagonist also attenuated status epilepticus induced vasogenic edema through the reduction of BBB disruption [182]. Moreover, the anti-edema action of the ET_B_-R antagonist was also shown in a cerebral ischemia model [183]. These findings indicate the possible use of ET_B_-R antagonists as an anti-vasogenic edema drug.

### 5.7. Glucocorticoid Receptor (GR)

Dexamethasone (DEX) is the most common synthetic glucocorticoid and leads to activation of GR. The beneficial effects of DEX for cerebral edema have been shown clinically. DEX administration can reduce brain edema in patients with intracranial tumors [184,185]. In experimental animals, the beneficial effects of DEX for brain edema in various brain insults have been indicated [186,187,188,189]. DEX is commonly used as an anti-inflammatory drug in the clinic. The inflammatory responses by cytokines and chemokines cause BBB breakdown and development of brain edema [190]. Thus, DEX may exert anti-edema action through attenuation of inflammatory responses. Moreover, the level of involvement in the regulation of BBB permeability is well known. DEX increases the levels of angiopoietin-1, which stabilizes the BBB structure and decreases the levels of VEGF in astrocytes and pericytes through GR activation [191]. Additionally, DEX has been shown to decrease the transmonolayer paracellular permeability through increases of tight junction-regulating proteins such as ZO-1 and occludin in cultured brain endothelial cells [192]. These findings imply that GR activation by DEX not only attenuates inflammatory responses but also stabilizes BBB, leading to a reduction of vasogenic brain edema.

## 6. Conclusions

Although brain edema is a fatal pathological state, the development of anti-edema drugs has been stagnant for decades. Understanding the pathogenesis of vasogenic and cytotoxic edema in various brain insults is important for the development of anti-edema drugs. By using experimental animal models for brain edema, key molecules involved in brain edema formation have been identified.

In experimental animals with traumatic brain injury and cerebral hemorrhage, BBB hyperpermeability-induced vasogenic edema is characteristic. As inducers of BBB hyperpermeability, VEGFs and MMP9 have been the primary focus. Increases in VEGFs and MMP9 have been observed, and the inhibition of VEGFs and MMP9 attenuates BBB disruption and reduces vasogenic edema in brain injury animals. Therefore, these inhibitors are candidate drugs for vasogenic edema. Moreover, ET_B_-R antagonists and DEX are confirmed to suppress VEGFs and MMP9 expressions in animals. The beneficial effects of ET_B_-R antagonists and DEX for BBB disruption and vasogenic edema are indicated in brain injury animals, and these are also expected to be a therapeutic strategy for vasogenic edema in the clinic (Table 1).

**Table 1:** Summary of candidate drugs for vasogenic and cytotoxic edema.

The Candidates of Anti-Edema Drugs	
Anti-vasogenic edema drugs	Anti-cytotoxic edema drugs
MMP inhibitors	KNCC1inhibitors (bumetanide)
VEGF inhibitors VEGF antibodies	SUR1-regulated NC_Ca-ATP_ inhibitors (glibenclamide)
ET_B_-R antagonists	–
Glucocorticoids (dexamethasone)	–

In cytotoxic edema animal models such as water intoxication and liver failure, disruptions of intra-extracellular Na^+^ balance and abnormal entry of water into cells cause cell swelling. AQP4 is indicated to be involved in the disturbance of intra-extra water balance and its expression fluctuates in brain injury animals. Although the involvement of AQP4 in cytotoxic edema is unambiguous, specific agents for AQP4 have not been found. The discovery of such agents would lead to progress in the development of anti-edema therapy. In the experimental brain edema model, NKCC1 and SUR1-regulated NC_Ca-ATP_ are also indicated to be responsible for cytotoxic edema formation. These functions are disturbed by brain injury, causing the induction of excessive Na^+^ entry into cells. The beneficial effects of bumetanide and glibenclamide for cytotoxic edema have been confirmed in experimental animals, and these medicines are good candidates to treat cytotoxic edema (Table 1). Both bumetanide and glibenclamide are used clinically, although adaptations for brain edema have not been established. Recently, the anti-edema effects of glyburide (Glibenclamide) were indicated by MRIs in patients with ischemic stroke [193] and further advancement is expected in the future.

The time-window and incidence of vasogenic and cytotoxic edema differ with the pathogenesis of brain injury. In cerebral ischemia, cytotoxic edema is observed in the early phase of injury, *i.e.*, within one day, whereas vasogenic edema is evoked after two to four days [11]. Thus, the timing of the administration of anti-vasogenic and cytotoxic edema drugs may be important for beneficial effects. Some of the candidate drugs introduced in this review are under clinical tests, and the discovery of novel anti-edema drugs among these is expected.
